# Strategies for Obtaining and Pruning Imputed Whole-Genome Sequence Data for Genomic Prediction

**DOI:** 10.3389/fgene.2019.00673

**Published:** 2019-07-17

**Authors:** Shaopan Ye, Ning Gao, Rongrong Zheng, Zitao Chen, Jinyan Teng, Xiaolong Yuan, Hao Zhang, Zanmou Chen, Xiquan Zhang, Jiaqi Li, Zhe Zhang

**Affiliations:** ^1^Guangdong Provincial Key Lab of Agro-Animal Genomics and Molecular Breeding, College of Animal Science, South China Agricultural University, Guangzhou, China; ^2^State Key Laboratory of Biocontrol, School of Life Sciences, Sun Yat-sen University, Guangzhou, China

**Keywords:** genomic prediction, imputed WGS data, LD-based marker pruning, GWAS, chickens

## Abstract

Genomic prediction with imputed whole-genome sequencing (WGS) data is an attractive approach to improve predictive ability with low cost. However, high accuracy has not been realized using this method in livestock. In this study, we imputed 435 individuals from 600K single nucleotide polymorphism (SNP) chip data to WGS data using different reference panels. We also investigated the prediction accuracy of genomic best linear unbiased prediction (GBLUP) using imputed WGS data from different reference panels, linkage disequilibrium (LD)-based marker pruning, and pre-selected variants based on Genome-wide association society (GWAS) results. Results showed that the imputation accuracies from 600K to WGS data were 0.873 ± 0.038, 0.906 ± 0.036, and 0.979 ± 0.010 for the internal, external, and combined reference panels, respectively. In most traits of chickens, the prediction accuracy of imputed WGS data obtained from the internal reference panel was greater than or equal to that of the combined reference panel; the external reference panel had the lowest prediction accuracy. Compared with 600K chip data, GBLUP with imputed WGS data had only a small increase (1–3%) in prediction accuracy. Using only variants selected from imputed WGS data based on GWAS results resulted in almost no increase for most traits and even increased the bias of the regression coefficient. The impact of the degree of LD of selected and remaining variants on prediction accuracy was different. For average daily gain (ADG), residual feed intake (RFI), intestine length (IL), and body weight in 91 days (BW91), the accuracy of GBLUP increased as the degree of LD of selected variants decreased, but the opposite relationship occurred for the remaining variants. But for breast muscle weight (BMW) and average daily feed intake (ADFI), the accuracy of GBLUP increased as the degree of LD of selected variants increased, and the degree of LD of remaining variants had a small effect on prediction accuracy. Overall, the optimal imputation strategy to obtain WGS data for genomic prediction should consider the relationship between selected individuals and target population individuals to avoid heterogeneity of imputation. LD-based marker pruning can be used to improve the accuracy of genomic prediction using imputed WGS data.

## Introduction

Whole genomic prediction (WGP) has been widely implemented in animal and plant breeding programs and human disease risk prediction. Over the past decade, the application of WGP was mainly based on SNP chip data. The rapid development of high-throughput sequencing technology has made it possible to apply WGP with whole-genome sequencing (WGS) data. Compared with SNP chip data, WGS data include whole genomic variants as well as causal mutations. Thus, WGP combined with WGS data were expected to lead to higher predictive ability. This expectation was confirmed by related simulation studies (Meuwissen and Goddard, 2010; Iheshiulor et al., 2016). However, using imputed WGS data to perform WGP has not led to the expected higher accuracies of genomic best linear unbiased prediction (GBLUP) or Bayesian methods in livestock. In cattle, van Binsbergen et al. (2015) found no benefit for genomic prediction using imputed WGS data compared with the HD chip data in a Holstein Friesian population. In addition, compared with the regular 54K chip data, imputed WGS data led to only a marginal increase in accuracy for nonreturn rate in heifers (NRH) and a decrease for stature and somatic cell score (SCS) in Brown Swiss cattle (Frischknecht et al., 2018). In pig, compared with 80K and 650K SNP chips, using imputed WGS data and BayesRC improved the prediction accuracies of loin muscle depth (LMD) and average daily gain (ADG); however, it resulted in decreased accuracy for ultrasound backfat depth (FAT) and average daily feed intake (ADFI) (Zhang et al., 2018). In chicken, there was little or no benefit using GBLUP with imputed WGS data compared with low-density or high-density (HD) chip data (Heidaritabar et al., 2016; Ni et al., 2017).

Many factors affect the prediction accuracy with imputed WGS data, such as the genetic architecture of traits, imputation errors, and linkage disequilibrium (LD). The genetic architecture of traits is the basis of genome prediction. If causal mutations all have low minor allele frequency (MAF), the accuracy of genomic prediction would be improved by up to 30% using sequence data (Druet et al., 2014). Thus, WGP with imputed WGS data is an attractive way to lead to higher predictive ability for low heritability traits. Imputed WGS data increases not only marker density to contain causative variants for traits but also imputation errors. Imputation errors decrease the accuracy of genomic predictions in the top segment and overestimate in the bottom segment, because imputation algorithms usually replace the missing haplotype with the most frequent haplotype observed in the reference panel (Pimentel et al., 2015). Selection of the imputation reference panel is very important for genomic prediction with imputed WGS data.

Currently, the imputation reference panels are always divided into three types: the internal reference panel, the external reference panel, and the combined reference panel. The combined reference panel has greater imputation accuracy, especially for low-frequency variants (Ye et al., 2018a). The imputed WGS data also include long-range LD within a breed, which may prevent the precise localization of quantitative trait nucleotides (QTN) (van Binsbergen et al., 2015). Moreover, most of these imputed variants were in imperfect LD with causative mutations. Therefore, it might be better to preselect fewer variants that are located closer to the QTN to improve the accuracy of genomic prediction (van den Berg et al., 2016).

Simulation studies have shown that only accurately preselected variants improve the accuracy of genomic prediction, due to including causative variants or a stronger LD with the causative mutations (Perez-Enciso et al., 2015; van den Berg et al., 2016). In multi-breeds, both reliability and accuracy were improved with selected sequence variants by GWAS (van den Berg et al., 2016; VanRaden et al., 2017; Teissier et al., 2018). However, in within-breed genomic prediction, selected sequence variants or weighting SNPs did not increase accuracy and even resulted in more bias (Veerkamp et al., 2016; Ni et al., 2017). Long-range LD within a breed may prevent the precise localization of QTN (van Binsbergen et al., 2015). Hence, it is necessary to carefully validate the degree of LD around significant variants for the implementation of WGP with imputed WGS data in within-breed genomic prediction.

In this study, we imputed 435 individuals from 600K chip data to WGS data using different reference panels and assessed the impact on the predictive ability of GBLUP. Then, we compared the prediction accuracy of GBLUP using different LD-based marker pruning and pre-selected variants based GWAS results. Additionally, these selected and remaining variants were pruned according to LD, and we explored the impact on prediction accuracy. These results of this study provide useful knowledge to obtain imputed WGS data and to execute genomic prediction within a livestock population.

## Materials and Methods

### Population and Phenotyping

The quality chicken population used in this study was derived from a yellow-feather dwarf broiler breed maintained for 25 generations by Wens Nanfang Poultry Breeding Co. Ltd. (Xinxing, P.R. China). This population included 1,600 birds (800 males and 800 females) and was the third batch of the 25th generation of this quality chicken population. These birds came from a mixture of full-sib and half-sib families from mating 30 males and 360 females of the 24th generation. After hatching, all birds were maintained in a closed building under controlled environmental conditions and provided with a standard diet until they were 4 weeks of age. The chickens were then randomly assigned to six pens by gender (three pens for males and three pens for females) for growth performance test from 5 to 13 weeks of age. They received food and water *ad libitum* during all stages. Finally, the birds were slaughtered at 91 days of age for carcass trait recording. For more details about this population, please refer to Xu et al. (2016) and Zhang et al. (2017).

Of these 1,600 birds, 1,338 were systematically phenotyped. During the growth performance test stage, body weights were recorded at 45, 49, 56, 63, 70, 77, 84, and 91 days of age (denoted as BW45, BW49, BW56, BW63, BW70, BW77, BW84, and BW91, respectively). The carcass weight (CW), eviscerated weight with giblets (EWG), eviscerated weight (EW), breast muscle weight (BMW), drumstick weight (DW), abdominal fat weight (AFW), abdominal fat percentage (AFP), gizzard weight (GW), and intestine length (IL) were measured at 91 days of age. Average daily gain (ADG) and feed conversion ratio (FCR) for each individual were calculated for the period from 45 to 84 days. The average daily feed intake (ADFI) and residual feed intake (RFI) were calculated with a previously reported formula (Xu et al., 2016): ADFI = b0 + b1 × MMBW + b2 × ADG + RFI, where b0 was the intercept, MMBW was mid-test body weight (MBW raised to the power of 0.75), and the MBW was the predicted body weight on day 21 of the test. b1 and b2 were the partial regression coefficients for MMBW and ADG, respectively, and RFI was the residual of the model. Detailed descriptive statistics for these 21 traits are in **Table S1**.

### Genotyping and Quality Control

After being systematically phenotyped, a total of 450 birds were randomly selected for genotyping with the 600K Affymetrix^®^ Axiom^®^ HD genotyping array (Kranis et al., 2013). These birds were 15 male parents and 435 male offspring. The average sire family size was 13.5 with a range of 7 to 23 in 435 genotyped male offspring. The 600K genotyping array contained 580,961 SNP probes across 28 autosomes, two linkage groups (LGE64 and LGE22C19W28_E50C23), and two sex chromosomes. After converting genome coordinates to a chicken reference genome (galGal5), we extracted 28 autosomes and a sex chromosome (chrZ) for further analyses. To process samples, genotyped SNPs with minor allele frequency (MAF) smaller than 0.5%, genotyping call rate smaller than 97%, and Hardy–Weinberg equilibrium test *p* value smaller than 1 × 10^−6^ were removed, resulting in 547,020 SNPs.

### Genotype Imputation and Quality Control

To check the effect of the different imputation reference panels on the predictive ability of imputed WGS data, the 600K chip data were imputed to WGS data with three reference panels (the internal, external, and combined reference panels). The internal reference panel was 24 key individuals of a yellow-feather dwarf broiler population. These 24 key individuals were selected from the 450 birds for re-sequencing by maximizing the expected genetic relationships (Ye et al., 2018b). The external reference panel was 311 birds with WGS data from diverse chicken breeds. They were downloaded from 10 BioProjects in ENA or NCBI. The BioProject numbers were PRJNA271711, PRJNA202483, PRJNA292383, PRJNA232548, PRJNA251505, PRJNA344300, PRJNA247952, PRJDB4092 (Ulfah et al., 2016), PRJNA241474, and PRJNA306389.

The combined reference panel was assembled using all individuals from the internal and external reference panels. Before pre-phasing, duplicate and monomorphic variants were excluded. In the target panel, we extracted overlapping sites between the 600K chip and reference panel and revised strand inconsistencies in A/T and C/G SNPs using conform-gt program (http://faculty.washington.edu/browning/conform-gt.html). Pre-phasing was executed in Beagle 4.1 with default analyses. All genotype imputations were also executed in Beagle 4.1 with default values (Browning and Browning, 2016). For all scenarios, we randomly masked 2% of SNPs from the target panel as the validation set before imputation, and then we calculated the correlation between the imputed genotypes and masked genotypes per individual. Detailed information about these reference panels is in our previous study (Ye et al., 2018a).

After we performed genotype imputation, we obtained three imputed WGS datasets from different reference panels. Quality control of the imputed WGS data was conducted using PLINK (Purcell et al., 2007) with the criteria of SNP call rate > 95%, individual call rate > 97%, MAF > 0.5%, and Hardy–Weinberg equilibrium *p* value > 1.0e−6. Finally, the remaining 435 individuals with SNPs obtained from combined (10,983,113), external (7,667,954), and internal reference panels (7,582,487) were used for further analysis.

### Pre-Selection and Classification of Whole-Genome Sequence Variants

The top variants (SNPs) were selected from the imputed WGS data based on their *p* value from GWAS results, as estimated in the training dataset using mixed linear model-based association analysis (MLMA) by GCTA software (Yang et al., 2011). All sequence variants after quality control were tested for associations. The model was

(Eq. 1) y=Xb+Zg+Sa+e

where y is a vector of phenotypic values of all individuals; X and Z are incidence matrices relating the fixed effects (overall mean) and the additive genetic values to the phenotypic records; b is the vector of fixed effects including pen effect; g is a vector of the genomic breeding values of all individuals; a is the additive effect of the candidate variants to be tested for association; **S** is a vector of the variants’ genotype indicator variable coded as 0, 1, or 2; and e is the residual term, $\text{e}∼\text{N(0},\sigma_{\text{e}}^{\text{2}}I\text{)}$. Genomic breeding values were assumed to be distributed as $\text{g}∼\text{N(0},\sigma_{\text{g}}^{\text{2}}G\text{)}$, where **G** is the genomic relationship matrix calculated using 600K chip data, as follows (VanRaden, 2008):

(Eq. 2) $$G=\;\frac{MM^{T}}{2\sum_{i=1}^{m}p_{i}(1-p_{i})}$$

where **M** is a matrix of centered genotypes, *m* is the number of markers, and *p* *_i_* is the minor allele frequency of SNP*_i_*

After GWAS analysis using imputed WGS data obtained from the internal reference panel, the *p* values from GWAS results were transformed with −log10, and variants were selected based on different *p* value cutoffs (from 2 to 5). The significant region of selected variants was defined as a region ± 50 kilobases (kb) from the physical location of an SNP. Then we extracted SNPs in significant regions from imputed WGS data using PLINK based on their corresponding genomic positions. After selection, the imputed WGS data were divided into selected variants and remaining variants.

## Genomic prediction

### Statistical Model

The breeding values of the 435 genotyped individuals were estimated with the standard GBLUP (VanRaden, 2008). The statistical model for the GBLUP approaches is

(Eq. 3) y=Xb+Zg+e

where y is a vector of phenotypic values; b is the vector of fixed effects including batch effect; g is a multivariate, normally distributed vector of additive genetic values for all individuals in the model; e is the residual term, $\text{e}∼\text{N(0},\sigma_{\text{e}}^{\text{2}}\text{I)}$; and X and Z are incidence matrices relating to the fixed effects (overall mean) and the additive genetic values to the phenotypic records. We assumed that the additive genetic value is $\text{g}∼\text{N(0},\sigma_{\text{g}}^{\text{2}}G\text{)}$, where G is a realized relationship matrix built with all or a subset of imputed WGS data as in VanRaden (2008). In Eq. 3, variance components were estimated using the “REML” algorithm via LDAK software (Speed and Balding, 2019). Given the dispersion matrices and the variance components, predictions of genetic values were obtained by solving the mixed model equations.

### Genomic Evaluation

In order to investigate the impact of the imputation reference panel, selected variants from GWAS results, and LD-based marker pruning on genomic prediction, four scenarios (denoted S1-S4) for genomic prediction were considered. Scenario 1 (S1) involved investigating the influence of the imputation reference panel on the predictive ability of imputed WGS data. We compared the predictive ability of 600K chip data and imputed WGS data from the internal reference panel, the external reference panel, and the combined reference panel. In scenario 2 (S2), the impact of LD-based marker pruning of chip data and imputed WGS data obtained from the internal reference panel on predictive ability was explored using GBLUP. Ten different R-squared cutoffs of LD (0.99, 0.9, 0.8, 0.7, 0.6, 0.5, 0.4, 0.3, 0.2, and 0.1) were set to prune markers of chip data and imputed WGS data. The summary of imputed WGS data before and after quality control is presented in **Table S3**.

Scenario 3 (S3) involved performing GBLUP with selected variants from different *p* value cutoffs (2–5) of GWAS results. In this scenario, the LD (R-squared > 0.99) was excluded for selection variants. The mean of selected variants from different *p*-value cutoffs of GWAS results is presented in **Table S4**.

Scenario 4 (S4) investigated the impact of the LD-based marker pruning of selected (or remaining) variants on genomic prediction. In this scenario, the variants of imputed WGS data were divided into selected and remaining variants using the *p*-value cutoffs [−log10(*p*) > 3] of GWAS results. Ten different R-squared cutoffs of LD (0.99, 0.9, 0.8, 0.7, 0.6, 0.5, 0.4, 0.3, 0.2, and 0.1) were set to prune remaining (or selected) variants. The number of selected or remaining variants with different R-squared cutoffs is presented in **Table S5**. Fixed R-squared of LD less than 0.1 was used to prune selected (or remaining) variants, and then the remaining (or selected) variants that were pruned with different LD-based marker pruning were merged. After a realized relationship matrix was built, only six traits were selected to perform GBLUP to improve computational efficiencies. These traits were ADG, IL, BMW, RFI, BW91, and ADFI. In this study, quality control of LD was conducted by PLINK in a 50-kb sliding window with 10 variants.

### Predictive Ability Evaluation

We used 10 replicates of a five-fold random cross-validation to assess the predictive ability of the different approaches for all traits. The variance components were estimated within the training set. Phenotypes of the validation set were treated as unknowns, and genetic values were predicted based on Eq. 3. The Pearson correlation between the predicted genetic values and the observed phenotypes corrected for fixed effect were defined as the prediction ability. In addition, the regression of the predicted genetic values and the observed phenotypes corrected for fixed effect was also calculated to assess the unbiasedness of predictions.

## Results

### Imputation Accuracy of Different Reference Panels

**Figure 1:**
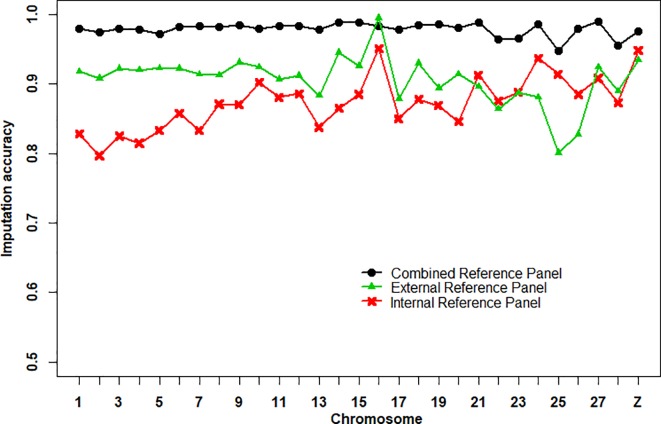
Average imputation accuracies of Beagle 4.1 using different reference panels per chromosome. The imputation accuracy was assessed by the correlation between imputed and masked true genotypes per SNPs.

The imputation accuracies of the internal reference panel (*n* = 24), the external reference panel (*n* = 311), and the combined reference panel (*n* = 335) from 600K chip data to WGS data per chromosome are shown in **Figure 1**. Imputation accuracy was assessed by correlations between the imputed and masked true genotypes per individual. We found that the average imputation accuracy of the combined reference panel (0.979 ± 0.010) was higher than that of the external reference panel (0.906 ± 0.036) and the internal reference panel (0.873 ± 0.038). At the chromosome level, the imputation accuracy of the combined reference panel was higher than that of the other reference panels, except for chromosome 16, where the imputation accuracy of the external reference panel was the highest. Compared with the other two reference panels, the imputation accuracy of the external reference panel on chr24, chr25, and chr26 was clearly lower. In addition, the numbers of variants in the combined reference panel (36,840,795 SNPs) and the external reference panel (36,715,962 SNPs) were 3.56-fold larger than in the internal reference panel (10,337,198 SNPs) (**Table S2**).

### Genomic Prediction With GBLUP Using Different Genotype Data

The prediction accuracy and regression coefficient of GBLUP of 21 traits in chicken using different genotype data are shown in **Table 1**. These genotype data included 600K chip data and imputed WGS datasets. These imputed WGS datasets were imputed from internal, external, and combined reference panels. In general, using GBLUP with imputed WGS data led to a small increase (1–3%) in prediction accuracy over the 600K chip data, except for ADG, BW45, and BW70. Comparing the prediction accuracy of GBLUP using imputed WGS data obtained from different reference panel, the internal or combined reference panel has higher prediction accuracy than the external reference panel, except for FCR. For most traits in chicken, the prediction accuracies for imputed WGS datasets obtained from the internal reference panel were greater than or equal to those of the combined reference panel, except for GW. In addition, regression coefficients were similar for the 600K chip data and imputed WGS data. Most regression coefficient values were considerably closer to 1, except for DW, GW, BW56, and BW63.

**Table 1:** Prediction accuracy and regression coefficients of 21 traits in chicken using genomic best linear unbiased prediction (GBLUP) with different genotype data.

Traits^1^	600K array^2^		WGS (internal)^3^		WGS (external)^4^		WGS (combined)^5^	
	${\text{r}}_{\left(\hat{\text{g}},\text{p}\right)}^{6}$	${\text{b}}_{\left(\hat{\text{g}},\text{p}\right)}^{7}$	${\text{r}}_{\left(\hat{\text{g}},\text{p}\right)}$	${\text{b}}_{\left(\hat{\text{g}},\text{p}\right)}$	${\text{r}}_{\left(\hat{\text{g}},\text{p}\right)}$	${\text{b}}_{\left(\hat{\text{g}},\text{p}\right)}$	${\text{r}}_{\left(\hat{\text{g}},\text{p}\right)}$	${\text{b}}_{\left(\hat{\text{g}},\text{p}\right)}$
ADG	**0.34 (0.01)**	0.98 (0.05)	0.33 (0.01)	0.99 (0.06)	0.33 (0.01)	1.0 2(0.06)	0.33 (0.01)	0.99 (0.06)
ADFI	0.40 (0.01)	1.01 (0.04)	**0.43 (0.01)**	1.00 (0.04)	0.42 (0.01)	1.02 (0.04)	0.42 (0.01)	1.0 0(0.04)
RFI	0.45 (0.01)	1.03 (0.04)	**0.47 (0.01)**	1.03 (0.04)	0.46 (0.01)	1.03 (0.04)	0.47 (0.01)	1.03 (0.04)
FCR	0.26 (0.01)	0.92 (0.06)	0.26 (0.01)	0.95 (0.06)	**0.27 (0.01)**	0.98 (0.06)	0.26 (0.01)	0.94 (0.06)
CW	0.30 (0.01)	1.01 (0.06)	**0.31 (0.01)**	0.99 (0.06)	0.29 (0.01)	1.01 (0.06)	0.30 (0.01)	0.99 (0.06)
EWG	0.28 (0.01)	0.97 (0.07)	**0.29 (0.01)**	0.99 (0.07)	0.27 (0.01)	1.00 (0.08)	0.28 (0.01)	0.99 (0.07)
EW	0.26 (0.01)	0.96 (0.07)	**0.27 (0.02)**	1.00 (0.08)	0.26 (0.02)	1.00 (0.08)	**0.27 (0.02)**	1.00 (0.08)
BMW	0.26 (0.02)	1.04 (0.09)	**0.27 (0.01)**	1.04 (0.08)	0.25 (0.01)	1.04 (0.08)	**0.27 (0.01)**	1.04 (0.08)
DW	0.20 (0.01)	1.10 (0.14)	**0.22 (0.01)**	1.11 (0.14)	0.20 (0.01)	1.12 (0.14)	0.21 (0.01)	1.10 (0.12)
AFW	0.36 (0.01)	1.04 (0.05)	0.36 (0.01)	1.02 (0.04)	0.36 (0.01)	1.04 (0.05)	0.36 (0.01)	1.02 (0.04)
AFP	0.32 (0.01)	1.00 (0.04)	**0.33 (0.01)**	1.00 (0.04)	0.32 (0.01)	0.98 (0.04)	**0.33 (0.01)**	1.00 (0.04)
GW	0.23 (0.01)	1.15 (0.10)	0.23 (0.01)	1.18 (0.10)	0.23 (0.01)	1.17 (0.10)	**0.24 (0.01)**	1.16 (0.10)
IL	0.24 (0.01)	1.09 (0.07)	**0.25 (0.01)**	1.07 (0.07)	0.24 (0.01)	1.08 (0.07)	**0.25 (0.01)**	1.07 (0.07)
BW45	0.28 (0.01)	1.06 (0.07)	**0.27 (0.01)**	1.06 (0.07)	0.25 (0.01)	1.09 (0.08)	**0.27 (0.01)**	1.05 (0.07)
BW49	0.27 (0.01)	1.07 (0.06)	**0.29 (0.01)**	1.06 (0.05)	0.26 (0.01)	1.08 (0.06)	0.28 (0.01)	1.05 (0.05)
BW56	0.29 (0.01)	1.19 (0.09)	**0.30 (0.01)**	1.18 (0.08)	0.27 (0.01)	1.19 (0.09)	**0.30 (0.01)**	1.18 (0.08)
BW63	0.26 (0.01)	1.22 (0.10)	**0.26 (0.02)**	1.23 (0.11)	0.24 (0.02)	1.28 (0.12)	**0.26 (0.02)**	1.25 (0.11)
BW70	0.26 (0.01)	1.06 (0.07)	**0.25 (0.01)**	1.06 (0.07)	0.24 (0.01)	1.08 (0.07)	**0.25 (0.01)**	1.04 (0.07)
BW77	0.29 (0.01)	0.99 (0.06)	**0.30 (0.01)**	1.03 (0.07)	0.29 (0.01)	1.06 (0.07)	0.29 (0.01)	1.02 (0.07)
BW84	0.32 (0.01)	1.05 (0.05)	**0.33 (0.01)**	1.05 (0.05)	0.31 (0.01)	1.07 (0.06)	0.32 (0.01)	1.06 (0.05)
BW91	0.30 (0.01)	1.01 (0.05)	**0.31 (0.01)**	0.98 (0.04)	0.30 (0.01)	1.00 (0.05)	**0.31 (0.01)**	0.98 (0.04)

^1^These traits were average daily gain (ADG), average daily feed intake (ADFI), residual feed intake (RFI), feed conversion ratio (FCR), carcass weight (CW), breast muscle weight (BMW), eviscerated weight with giblets (EWG), eviscerated weight (EW), drumstick weight (DW), abdominal fat weight (AFW), abdominal fat percentage (AFP), gizzard weight (GW), intestine length (IL), body weight in 45 days (BW45), body weight in 49 days (BW49), body weight in 56 days (BW56), body weight in 63 days (BW63), body weight in 70 days (BW70), body weight in 77 days (BW77), body weight in 84 days (BW84), and body weight in 91 days (BW91).

^2^600K array: the 600K Affymetrix^®^ Axiom^®^ HD genotyping array.

^3^WGS (internal): the imputed whole-genome sequencing data obtained from internal reference panel.

^4^WGS (external): the imputed whole-genome sequencing data obtained from external reference panel.

^5^WGS (combined): the imputed whole-genome sequencing data obtained from combined reference panel.

^6^${\text{r}}_{\left(\hat{\text{g}},\text{p}\right)}$: the Pearson correlation between the predicted genetic values (ĝ) and the observed phenotypes (p) corrected for fixed effect. Means and standard errors (in parentheses) of ${\text{r}}_{\left(\hat{\text{g}},\text{p}\right)}$ are shown in the table. Bold font was used to represent values that were higher than others.

^7^${\text{b}}_{\left(\hat{\text{g}},\text{p}\right)}$: regression of the predicted genetic values (ĝ) and the observed phenotypes (p) corrected for fixed effect. Means and standard errors (in parentheses) of regression coefficients are shown in the table.

### Impact of Linkage Disequilibrium (LD)-Based Marker Pruning on Genomic Prediction

**Figure 2:**
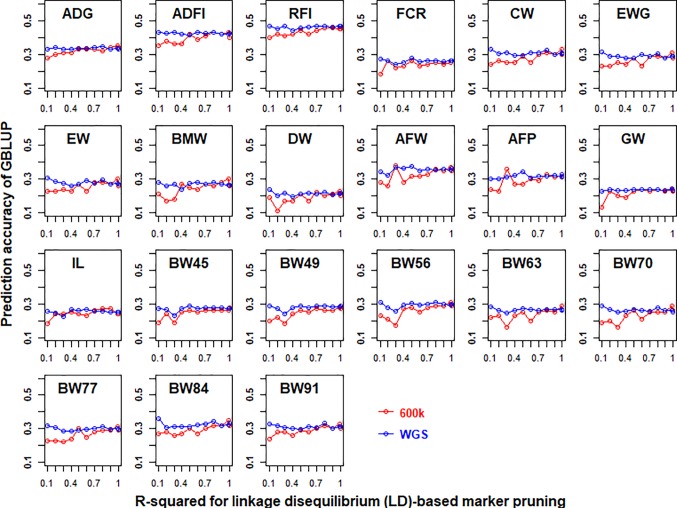
Impact of linkage disequilibrium (LD)-based marker pruning on the predictive ability of imputed whole-genome sequencing (WGS) and chip data. Different R-squared cutoffs of LD (0.99, 0.9, 0.8, 0.7, 0.6, 0.5, 0.4, 0.3, 0.2, and 0.1) were used to prune markers of imputed WGS and chip data. The predictive ability was assessed by the Pearson correlation between the predicted genetic values and the observed phenotypes corrected for fixed effect per trait. These traits were average daily gain (ADG), average daily feed intake (ADFI), residual feed intake (RFI), feed conversion ratio (FCR), carcass weight (CW), breast muscle weight (BMW), eviscerated weight with giblets (EWG), eviscerated weight (EW), drumstick weight (DW), abdominal fat weight (AFW), abdominal fat percentage (AFP), gizzard weight (GW), intestine length (IL), body weight in 45 days (BW45), body weight in 49 days (BW49), body weight in 56 days (BW56), body weight in 63 days (BW63), body weight in 70 days (BW70), body weight in 77 days (BW77), body weight in 84 days (BW84), and body weight in 91 days (BW91).

The prediction accuracies of GBLUP of 21 traits in chicken using both 600K chip data and imputed WGS data that had different LD-based markers pruning are shown in **Figure 2**. In general, LD-based marker pruning increased prediction accuracies for 600K chip data (1–4%, except for BW45) and imputed WGS data (0.1–3.7%) compared with whole genotype data. For 600K chip data, the prediction accuracies of most traits using LD-based marker pruning (R-squared < 0.99) reached the highest level, and the prediction accuracies tended to decrease as the R-squared cutoff decreased. In contrast, the prediction accuracies of most traits using imputed WGS data were the highest when the R-squared cutoff (0.1) of LD was used to prune markers. The number of markers of imputed WGS data and 600K chip data with different R-squared cutoffs of LD is in **Table S3**.

### Genomic Prediction With Selected Variants From GWAS Results

**Figure 3:**
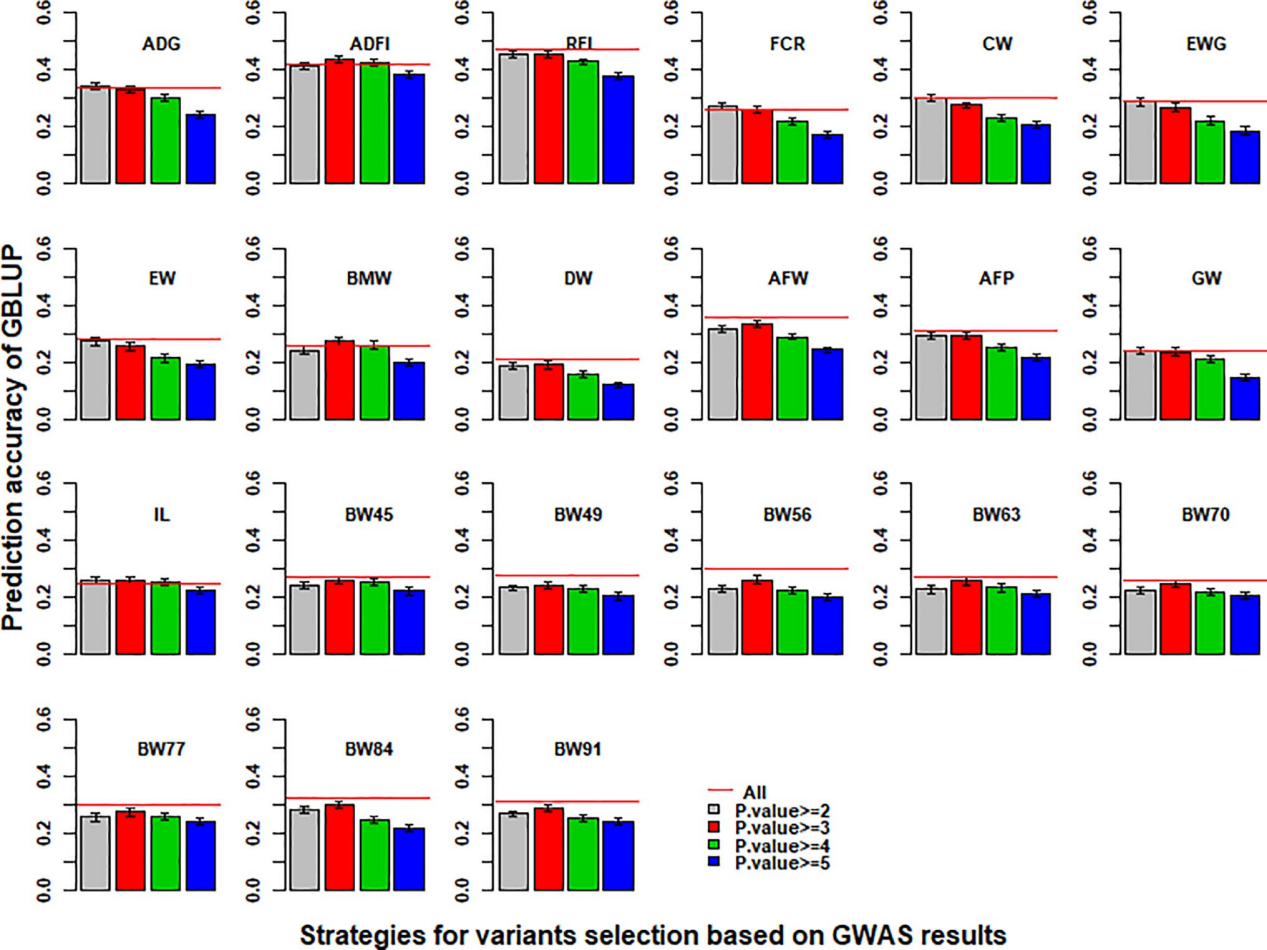
Impact of pre-selected variants on the predictive ability of GBLUP using imputed WGS data. Different *p*-value cutoffs from 2 to 5 were used to select variants from imputed WGS data based on GWAS results for GBLUP. The red line was the prediction accuracy of GBLUP with all markers of imputed WGS data. The predictive ability was assessed by the Pearson correlation between the predicted genetic values and the observed phenotypes corrected for fixed effect(s) per trait. These traits were average daily gain (ADG), average daily feed intake (ADFI), residual feed intake (RFI), feed conversion ratio (FCR), carcass weight (CW), breast muscle weight (BMW), eviscerated weight with giblets (EWG), eviscerated weight (EW), drumstick weight (DW), abdominal fat weight (AFW), abdominal fat percentage (AFP), gizzard weight (GW), intestine length (IL), body weight in 45 days (BW45), body weight in 49 days (BW49), body weight in 56 days (BW56), body weight in 63 days (BW63), body weight in 70 days (BW70), body weight in 77 days (BW77), body weight in 84 days (BW84), and body weight in 91 days (BW91).

With the use of variants selected from imputed WGS data based on the *p* value of GWAS results to perform GBLUP, the prediction accuracies of 21 traits in chicken are shown in **Figure 3**. Compared with the imputed WGS data, the best prediction accuracy of GBLUP with selected variants had almost no increase for most traits except for ADG, ADFI, BMW, FCR, and IL and even increased the bias for regression coefficients (**Table S6**). In addition, the prediction accuracy of GBLUP with selected variants was the highest when the cutoff of *p* value of GWAS results of more than 2 or 3 was used to select variants for genomic prediction.

### Impact of the Linkage Disequilibrium (LD)-Based Marker Pruning of Selected or Remaining Variants on Genomic Prediction

**Figure 4:**
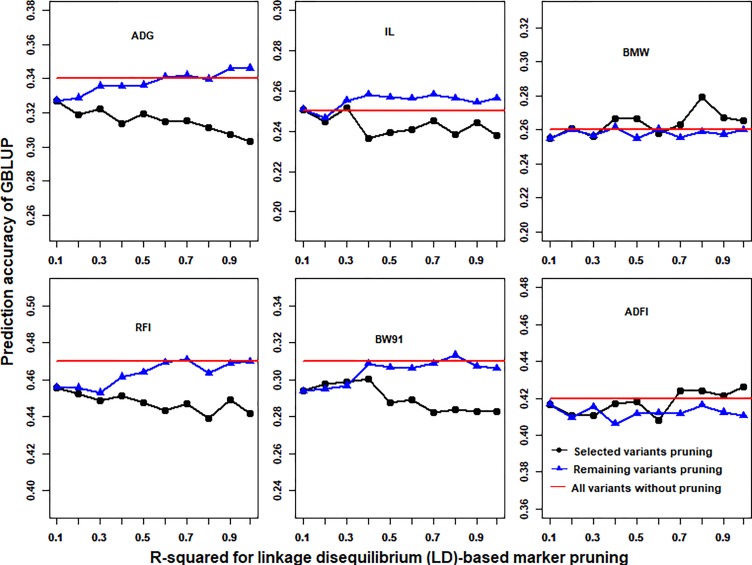
Impact of the linkage disequilibrium (LD)-based marker pruning of selected or remaining variants on prediction accuracy. R-squared of LD less than 0.1 was fixed to prune selected (or remaining) variants, and then the remaining (or selected) variants that were pruned with different R-squared cutoffs of LD (0.99, 0.9, 0.8, 0.7, 0.6, 0.5, 0.4, 0.3, 0.2, and 0.1) for GBLUP were merged. The red line is the prediction accuracy of GBLUP with all markers of imputed WGS data. The predictive ability was assessed by the Pearson correlation between the predicted genetic values and the observed phenotypes corrected for fixed effect(s) per trait. These traits were average daily gain (ADG), intestine length (IL), breast muscle weight (BMW), residual feed intake (RFI), body weight in 91 days (BW91), and average daily feed intake (ADFI).

With the use of the *p*-value cutoffs (−log10(*p*) > 3) of GWAS results, the imputed WGS data were divided into selected and remaining variants. R-squared of LD less than 0.1 was fixed to prune selected (or remaining) variants, and then the remaining (or selected) variants that were pruned with different R-squared cutoffs of LD (0.99, 0.9, 0.8, 0.7, 0.6, 0.5, 0.4, 0.3, 0.2, and 0.1) for GBLUP were merged. The prediction accuracies of GBLUP of six traits in chicken using different LD-based marker pruning of selected or remaining variants are shown in **Figure 4**. In general, the LD-based marker pruning improved the prediction accuracy compared with WGS data without LD-based marker pruning. The impact of the degree of LD of selected and remaining variants on prediction accuracy was different. For some traits (ADG, RFI, IL, and BW91), the accuracy of GBLUP was increased as the degree of LD of selected variants decreased, but in contrast to remaining variants. However, in both BMW and ADFI, the accuracy of GBLUP was increased as the degree of LD of selected variants increased, and the degree of LD of remaining variants had a small effect on prediction accuracy. Additionally, the average number of selected and remaining variants was increased as the R-squared cutoffs increased (**Table S5**).

## Discussion

Genomic prediction with imputed WGS data is an attractive approach to improve predictive ability with low cost. However, the expected higher accuracies of this method have not been realized in livestock. In the present study, we determined the impact of genotype imputation reference panels, LD-based marker pruning, and preselected variants on prediction accuracy. Overall, imputed WGS data obtained from the internal reference panel achieved higher predictive ability than did other reference panels. Preselecting variants from GWAS results and performing careful LD-based marker pruning would improve the prediction accuracy of imputed WGS data.

### Genomic Prediction With Imputed Whole-Genome Sequence Data

The expectations for higher accuracies have not been realized using WGP with imputed WGS data in livestock. Our results showed that simply using WGS data resulted in a small increase (1–3%) in prediction accuracy and that, in some traits, it even resulted in lower accuracies than using 600K chip data. These results are in agreement with previous studies that showed a small or no added benefit of using WGS data for WGP (van Binsbergen et al., 2015; Heidaritabar et al., 2016; Ni et al., 2017). The effect of the causal markers could not be evaluated precisely because a large number of non-causal markers were in linkage disequilibrium with causal mutations (van Binsbergen et al., 2015). In a simulation study, increasing the numbers of SNPs from 54K to 500K without including the causative mutations only increased prediction accuracy by 1.6% (VanRaden et al., 2011). Additionally, GBLUP is an infinitesimal model that assumes all markers have the same contribution to the trait. If a trait had no major QTL, the accuracy of genomic prediction could not be improved with WGS data, unless a large reference population is used (Clark et al., 2011). Similar results were also found where if the allele frequency spectrum of the causative mutations followed a neutral distribution, the advantage of sequence data over SNP panels was small, whereas with the causal mutations with very low frequencies, this advantage was up to 30% (Druet et al., 2014).

### Genotype Imputation

An obvious improvement in imputation accuracy was observed using a combined reference panel for genotype imputation (**Figure 1**). Similar results were found in a previous study (Mitt et al., 2017; Ye et al., 2018a). Higher imputation accuracy was expected lead to higher prediction accuracy of WGP (Moghaddar et al., 2015). However, the expectations for higher predictive ability were not realized in this study. We found that WGP with imputed WGS data obtained from the internal reference panel achieved higher prediction accuracy than did the other two reference panels, although the average imputation accuracy was lowest (**Figure 1**, **Table 1**). This may be caused by similar imputation errors between related individuals and because some genetic relationships were captured in the WGP model (Weigel et al., 2010). It may also be due to an undesirable association between the reference panel and target population, resulting in worse prediction accuracy. For example, the prediction accuracy of imputed WGS data obtained from the external reference panel was the worst (**Table 1**), due to the large number of undesirable associations between the external reference panel and target population. Therefore, it is necessary to consider the relationship between selected individuals and target population individuals to avoid heterogeneity of imputation, especially when using external population individuals to expand the genotype imputation reference panel size.

### Selected Variants From Imputed Whole-Genome Sequence Data

Variants selected from imputed WGS data can be used to improve the accuracy of genomic prediction (Raymond et al., 2018; Teissier et al., 2018). In this study, the accuracy of genomic prediction with selected variants had almost no increase or only a small increase (**Figure 3**). A higher −log10(*p*) threshold resulted in less selection variants (**Table S4**) and poor predictive ability (**Figure 3**), likely because the GBLUP model had a greater tendency to overfit in the training set using only a few significant selection variants. The issue could be overcome by adding smaller numbers of selected sequence variants to low- or high-density chip data, consistent with previous studies (Brondum et al., 2015; VanRaden et al., 2017; Raymond et al., 2018). Additionally, it was difficult to detect causal variants based on GWAS due to the large number of variants, and the high LD between variants.

### Linkage Disequilibrium (LD)-Based Marker Pruning

LD plays an important role in genomic selection (Meuwissen et al., 2001). In this study, results showed that stronger LD resulted in higher accuracy of genomic predictions when GBLUP was performed with 600K chip data (**Figure 2**). A similar result was also found in a previous study (Calus et al., 2008). However, with the use of imputed WGS data, lower LD resulted in higher accuracy of genomic predictions (**Figure 2**). A possible reason is that imputed WGS data include causal mutations and a large number of variants were in imperfect LD between causal mutations, which would reduce prediction reliability (de Los Campos et al., 2013). Furthermore, the impact of LD patterns was different in selection and remaining variants on genomic prediction (**Figure 4**). This may be due to the different genetic architecture of traits. For these traits (ADG, RFI, IL, and BW91), the accuracy of GBLUP was increased as the degree of LD of selected variants decreased, but the opposite relationship occurred for remaining variants. This means that these selected variants included the causal mutations of traits and the accuracy of genomic prediction could be improved by reducing markers that were in imperfect LD between causal mutations. Additionally, SNPs used for genomic selection not only capture LD between SNP and QTL but capture family relationships among individuals as well (Habier et al., 2010). The stronger LD of remaining variants reflected the family relationships among individuals and had a large effect on the accuracy of genomic predictions (Wientjes et al., 2013). For BMW and ADFI, the accuracy of GBLUP increased as the degree of LD of selected variants increased, and the degree of LD of remaining variants had only a small effect on prediction accuracy (**Figure 4**). It may be that only a small proportion of SNPs have a large effect on these traits. The stronger LD of selection variants seems likely to affect the major QTL for GBLUP and results in higher accuracy of genomic prediction (Zhang et al., 2014).

## Data Availability

Publicly available datasets were analyzed in this study. This data can be found here: http://www.animalgenome.org/repository/pub/SCAU2016.0217/

The 600 K chip data of these 450 birds has been uploaded to the figshare repository (https://doi.org/10.6084/m9.figshare.8295299).

## Ethics Statement

All animal care and handling procedures conformed to the Animal Care Committee of South China Agriculture University (Guangzhou, People’s Republic of China). Animals involved in this study were humanely sacrificed as necessary to ameliorate their suffering.

## Author Contributions

SY, ZZ, and JL conceived the study, designed the project, and helped draft the manuscript. XZ provided the chicken dataset. SY and RZ analyzed the sequencing data and finished the genotype imputation. SY and NG performed genomic prediction and analyzed the accuracy. ZC, JT, XY, HZ, and ZC participated in the design and contributed to the drafting of the manuscript. All authors read and approved the manuscript.

## Funding

This work was supported by the National Natural Science Foundation of China (31772556), the earmarked fund for China Agriculture Research System (CARS-35, CARS-41), and the Special Program for Applied Research on Super Computation of the NSFC-Guangdong Joint Fund (the second phase) under Grant No. U1501501.

## Conflict of Interest Statement

The authors declare that the research was conducted in the absence of any commercial or financial relationships that could be construed as a potential conflict of interest.

## References

[B1] Brondum R. F. Su G. Janss L. Sahana G. Guldbrandtsen B. Boichard D. (2015). Quantitative trait loci markers derived from whole genome sequence data increases the reliability of genomic prediction. J. Dairy Sci. 98 (6), 4107–4116. 10.3168/jds.2014-9005 25892697

[B2] Browning B. Browning S. (2016). Genotype imputation with millions of reference samples. Am. J. Hum. Genet. 98 (1), 116–126. 10.1016/j.ajhg.2015.11.020 26748515 PMC4716681

[B3] Calus M. P. Meuwissen T. H. de Roos A. P. Veerkamp R. F. (2008). Accuracy of genomic selection using different methods to define haplotypes. Genetics 178 (1), 553–561. 10.1534/genetics.107.080838 18202394 PMC2206101

[B4] Clark S. A. Hickey J. M. van der Werf J. H. (2011). Different models of genetic variation and their effect on genomic evaluation. Genet. Sel. Evol. 43, 18. 10.1186/1297-9686-43-18 21575265 PMC3114710

[B5] de Los Campos G. Vazquez A. I. Fernando R. Klimentidis Y. C. Sorensen D. (2013). Prediction of complex human traits using the genomic best linear unbiased predictor. PLoS Genet. 9 (7), e1003608. 10.1371/journal.pgen.1003608 23874214 PMC3708840

[B6] Druet T. Macleod I. M. Hayes B. J. (2014). Toward genomic prediction from whole-genome sequence data: impact of sequencing design on genotype imputation and accuracy of predictions. Heredity 112 (1), 39–47. 10.1038/hdy.2013.13 23549338 PMC3860159

[B7] Frischknecht M. Meuwissen T. H. E. Bapst B. Seefried F. R. Flury C. Garrick D. (2018). Short communication: genomic prediction using imputed whole-genome sequence variants in Brown Swiss Cattle. J. Dairy Sci. 101 (2), 1292–1296. 10.3168/jds.2017-12890 29153527

[B8] Habier D. Tetens J. Seefried F. R. Lichtner P. Thaller G. (2010). The impact of genetic relationship information on genomic breeding values in German Holstein cattle. Genet. Sel. Evol. 42, 5. 10.1186/1297-9686-42-5 20170500 PMC2838754

[B9] Heidaritabar M. Calus M. P. Megens H. J. Vereijken A. Groenen M. A. Bastiaansen J. W. (2016). Accuracy of genomic prediction using imputed whole-genome sequence data in white layers. J. Anim. Breed Genet. 133 (3), 167–179. 10.1111/jbg.12199 26776363

[B10] Iheshiulor O. O. Woolliams J. A. Yu X. Wellmann R. Meuwissen T. H. (2016). Within- and across-breed genomic prediction using whole-genome sequence and single nucleotide polymorphism panels. Genet. Sel. Evol. 48 (1), 15. 10.1186/s12711-016-0193-1 26895843 PMC4759725

[B11] Kranis A. Gheyas A. A. Boschiero C. Turner F. Yu L. Smith S. (2013). Development of a high density 600K SNP genotyping array for chicken. BMC Genomics 14 (1), 59. 10.1186/1471-2164-14-59 23356797 PMC3598943

[B12] Meuwissen T. Goddard M. (2010). Accurate prediction of genetic values for complex traits by whole-genome resequencing. Genetics 185 (2), 623–631. 10.1534/genetics.110.116590 20308278 PMC2881142

[B13] Meuwissen T. H. Hayes B. J. Goddard M. E. (2001). Prediction of total genetic value using genome-wide dense marker maps. Genetics 157 (4), 1819–1829. 11290733 10.1093/genetics/157.4.1819 PMC1461589

[B14] Mitt M. Kals M. Parn K. Gabriel S. B. Lander E. S. Palotie A. (2017). Improved imputation accuracy of rare and low-frequency variants using population-specific high-coverage WGS-based imputation reference panel. Eur. J. Hum. Genet. 25 (7), 869–876. 10.1038/ejhg.2017.51 28401899 PMC5520064

[B15] Moghaddar N. Gore K. P. Daetwyler H. D. Hayes B. J. van der Werf J. H. (2015). Accuracy of genotype imputation based on random and selected reference sets in purebred and crossbred sheep populations and its effect on accuracy of genomic prediction. Genet. Sel. Evol. 47, 97. 10.1186/s12711-015-0175-8 26694131 PMC4688977

[B16] Ni G. Cavero D. Fangmann A. Erbe M. Simianer H. (2017). Whole-genome sequence-based genomic prediction in laying chickens with different genomic relationship matrices to account for genetic architecture. Genet. Sel. Evol. 49 (1), 8. 10.1186/s12711-016-0277-y 28093063 PMC5238523

[B17] Perez-Enciso M. Rincon J. C. Legarra A. (2015). Sequence- vs. chip-assisted genomic selection: accurate biological information is advised. Genet. Sel. Evol. 47, 43. 10.1186/s12711-015-0117-5 25956961 PMC4424891

[B18] Pimentel E. C. Edel C. Emmerling R. Gotz K. U. (2015). How imputation errors bias genomic predictions. J. Dairy Sci. 98 (6), 4131–4138. 10.3168/jds.2014-9170 25841966

[B19] Purcell S. Neale B. Todd-Brown K. Thomas L. Ferreira M. A. Bender D. (2007) PLINK: a tool set for whole-genome association and population-based linkage analyses. Am. J. Hum. Genet. 81 (3), 559–575. 17701901 10.1086/519795 PMC1950838

[B20] Raymond B. Bouwman A. C. Wientjes Y. C. J. Schrooten C. Houwing-Duistermaat J. Veerkamp R. F. (2018). Genomic prediction for numerically small breeds, using models with pre-selected and differentially weighted markers. Genet. Sel. Evol. 50 (1), 49. 10.1186/s12711-018-0419-5 30314431 PMC6186145

[B21] Speed D. Balding D. J. (2019). SumHer better estimates the SNP heritability of complex traits from summary statistics. Nat. Genet. 51 (2), 277–284. 10.1038/s41588-018-0279-5 30510236 PMC6485398

[B22] Teissier M. Sanchez M. P. Boussaha M. Barbat A. Hoze C. Robert-Granie C. (2018). Use of meta-analyses and joint analyses to select variants in whole genome sequences for genomic evaluation: an application in milk production of French dairy cattle breeds. J. Dairy Sci. 101 (4), 3126–3139. 10.3168/jds.2017-13587 29428760

[B23] Ulfah M. Kawahara-Miki R. Farajalllah A. Muladno M. Dorshorst B. Martin A. (2016). Genetic features of red and green junglefowls and relationship with Indonesian native chickens Sumatera and Kedu Hitam. BMC Genomics 17 (1), 320. 10.1186/s12864-016-2652-z 27142387 PMC4855759

[B24] van Binsbergen R. Calus M. P. Bink M. C. van Eeuwijk F. A. Schrooten C. Veerkamp R. F. (2015). Genomic prediction using imputed whole-genome sequence data in Holstein Friesian cattle. Genet. Sel. Evol. 47, 71. 10.1186/s12711-015-0149-x 26381777 PMC4574568

[B25] van den Berg I. Boichard D. Guldbrandtsen B. Lund M. S. (2016). Using sequence variants in linkage disequilibrium with causative mutations to improve across-breed prediction in dairy cattle: a simulation study. G3-Genes Genom. Genet. 6 (8), 2553–2561. 10.1534/g3.116.027730 PMC4978908 27317779

[B26] VanRaden P. M. (2008). Efficient methods to compute genomic predictions. J. Dairy Sci. 91 (11), 4414–4423. 10.3168/jds.2007-0980 18946147

[B27] VanRaden P. M. O’Connell J. R. Wiggans G. R. Weigel K. A. (2011). Genomic evaluations with many more genotypes. Genet. Sel. Evol. 43, 10. 10.1186/1297-9686-43-10 21366914 PMC3056758

[B28] VanRaden P. M. Tooker M. E. O’Connell J. R. Cole J. B. Bickhart D. M. (2017). Selecting sequence variants to improve genomic predictions for dairy cattle. Genet. Sel. Evol. 49 (1), 32. 10.1186/s12711-017-0307-4 28270096 PMC5339980

[B29] Veerkamp R. F. Bouwman A. C. Schrooten C. Calus M. P. (2016). Genomic prediction using preselected DNA variants from a GWAS with whole-genome sequence data in Holstein-Friesian cattle. Genet. Sel. Evol. 48 (1), 95. 10.1186/s12711-016-0274-1 27905878 PMC5134274

[B30] Weigel K. A. de Los Campos G. Vazquez A. I. Rosa G. J. Gianola D. Van Tassell C. P. (2010). Accuracy of direct genomic values derived from imputed single nucleotide polymorphism genotypes in Jersey cattle. J. Dairy Sci. 93 (11), 5423–5435. 10.3168/jds.2010-3149 20965358

[B31] Wientjes Y. C. Veerkamp R. F. Calus M. P. (2013). The effect of linkage disequilibrium and family relationships on the reliability of genomic prediction. Genetics 193 (2), 621–631. 10.1534/genetics.112.146290 23267052 PMC3567749

[B32] Xu Z. Ji C. Zhang Y. Zhang Z. Nie Q. Xu J. (2016). Combination analysis of genome-wide association and transcriptome sequencing of residual feed intake in quality chickens. BMC Genomics 17 (1), 594. 10.1186/s12864-016-2861-5 27506765 PMC4979145

[B33] Yang J. Lee S. H. Goddard M. E. Visscher P. M. (2011). GCTA: a tool for genome-wide complex trait analysis. Am. J. Hum. Genet. 88 (1), 76–82. 10.1016/j.ajhg.2010.11.011 21167468 PMC3014363

[B34] Ye S. Yuan X. Huang S. Zhang H. Chen Z. Li J. (2018a). Comparison of genotype imputation strategies using a combined reference panel for chicken population. Animal 13 (6), 1119–1126. 10.1017/S1751731118002860 30370890

[B35] Ye S. P. Yuan X. L. Lin X. R. Gao N. Luo Y. Y. Chen Z. M. (2018b). Imputation from SNP chip to sequence: a case study in a Chinese indigenous chicken population. J. Anim. Sci. Biotechnol. 9 (1), 30. 10.1186/s40104-018-0241-5 29581880 PMC5861640

[B36] Zhang C. Kemp R. A. Stothard P. Wang Z. Boddicker N. Krivushin K. (2018). Genomic evaluation of feed efficiency component traits in Duroc pigs using 80K, 650K and whole-genome sequence variants. Genet. Sel. Evol. 50 (1), 14. 10.1186/s12711-018-0387-9 29625549 PMC5889553

[B37] Zhang Z. Ober U. Erbe M. Zhang H. Gao N. He J. (2014). Improving the accuracy of whole genome prediction for complex traits using the results of genome wide association studies. PLoS One 9 (3), e93017. 10.1371/journal.pone.0093017 24663104 PMC3963961

[B38] Zhang Z. Xu Z. Q. Luo Y. Y. Zhang H. B. Gao N. He J. L. (2017). Whole genomic prediction of growth and carcass traits in a Chinese quality chicken population. J. Anim .Sci. 95 (1), 72–80. 10.2527/jas.2016.0823 28177394

