# Supplementary material for: Strategies for Obtaining and Pruning Imputed Whole-Genome Sequence Data for Genomic Prediction
Source: Front Genet. 2019 Jul 17;10:673. doi: 10.3389/fgene.2019.00673 (PMC6650575; doi:10.3389/fgene.2019.00673)
Supplement: Supplementary file 1 [file Table_1.docx]

Supplementary Material

# Supplementary Data

The Affymetrix 600 K genotyping data are available at the animal genome website (http://www.animalgenome.org/repository/pub/SCAU2016.0217/). The raw phenotype data and genotype imputation reference panel are available from the corresponding author on reasonable request.

# Supplementary Tables

**Supplementary Table S1. Descriptive statistics of the 21 traits in chicken**

| Traits^1^ | N | Min | Max | Mean | SD |
| --- | --- | --- | --- | --- | --- |
|  |  |  |  |  |  |
| ADG, g/d | 435 | 15.3 | 44.6 | 29.0 | 4.7 |
| ADFI, g/d | 435 | 69.8 | 149.9 | 109.7 | 14.5 |
| FCR | 435 | 2.9 | 6.9 | 3.9 | 0.5 |
| RFI, % | 435 | -25.5 | 25.5 | 0.2 | 8.4 |
| CW, g | 423 | 983.7 | 2188.0 | 1623.3 | 200.7 |
| EWG, g | 430 | 915.8 | 2007.7 | 1486.5 | 187.5 |
| EW, g | 430 | 732.8 | 1704.0 | 1237.2 | 160.2 |
| BMW, g | 430 | 51.2 | 190.0 | 101.1 | 16.8 |
| DW, g | 430 | 112.0 | 328.0 | 216.3 | 31.7 |
| AFW, g | 430 | 3.0 | 124.0 | 48.2 | 19.9 |
| AFP, % | 430 | 0.3 | 9.3 | 3.8 | 1.4 |
| GW, g | 426 | 8.0 | 56.0 | 31.6 | 6.3 |
| IL, cm | 434 | 84.0 | 226.0 | 165.6 | 15.5 |
| BW49, g | 435 | 570.0 | 1332.0 | 925.7 | 139.2 |
| BW56, g | 434 | 736.0 | 1607.0 | 1153.9 | 158.2 |
| BW63, g | 432 | 831.0 | 1831.0 | 1346.6 | 170.5 |
| BW70, g | 435 | 1096.0 | 2114.0 | 1560.7 | 184.1 |
| BW77, g | 434 | 1190.0 | 2319.0 | 1761.7 | 204.1 |
| BW84, g | 433 | 1247.0 | 2591.0 | 1916.7 | 228.5 |
| BW91, g | 416 | 1093.0 | 2392.0 | 1816.1 | 222.0 |

^1^ These traits were average daily gain (ADG), average daily feed intake (ADFI), residual feed intake (RFI), feed conversion ratio (FCR), carcass weight (CW), breast muscle weight (BMW), eviscerated weight with giblets (EWG), eviscerated weight (EW), drumstick weight (DW), abdominal fat weight (AFW), abdominal fat percentage (AFP), gizzard weight (GW), intestine length (IL), body weight in 45 days (BW45), body weight in 49 days (BW49), body weight in 56 days (BW56), body weight in 63 days (BW63), body weight in 70 days (BW70), body weight in 77 days (BW77), body weight in 84 days (BW84), and body weight in 91 days (BW91).

**Supplementary Table S2. The number of markers in different reference and target panels in different genotype imputation**

| Chr. | Combine^1^ | |  | Internal^2^ | |  | External^3^ | |
| --- | --- | --- | --- | --- | --- | --- | --- | --- |
|  | Tar.snp^4^ | Ref.snp^5^ |  | Tar.snp | Ref.snp |  | Tar.snp | Ref.snp |
| 1 | 86,634 | 7,158,664 |  | 70,005 | 2,092,043 |  | 86,277 | 7,133,839 |
| 2 | 54,270 | 5,407,938 |  | 44,330 | 1,593,990 |  | 54,010 | 5,388,520 |
| 3 | 48,458 | 4,079,325 |  | 39,134 | 1,188,824 |  | 48,245 | 4,065,302 |
| 4 | 36,781 | 3,386,519 |  | 30,270 | 1,008,056 |  | 36,655 | 3,374,735 |
| 5 | 26,419 | 2,210,233 |  | 21,668 | 643,710 |  | 26,341 | 2,203,838 |
| 6 | 19,065 | 1,479,613 |  | 15,092 | 429,311 |  | 18,976 | 1,475,225 |
| 7 | 18,632 | 1,369,206 |  | 14,972 | 397,780 |  | 18,546 | 1,364,900 |
| 8 | 14,884 | 1,047,018 |  | 11,668 | 278,932 |  | 14,837 | 1,044,541 |
| 9 | 15,740 | 944,278 |  | 12,570 | 263,125 |  | 15,668 | 942,245 |
| 10 | 16,081 | 756,469 |  | 12,296 | 212,718 |  | 16,005 | 754,212 |
| 11 | 11,717 | 690,058 |  | 9,202 | 187,616 |  | 11,678 | 687,974 |
| 12 | 12,633 | 784,474 |  | 9,601 | 212,725 |  | 12,600 | 782,467 |
| 13 | 9,605 | 703,595 |  | 7,869 | 206,695 |  | 9,572 | 701,615 |
| 14 | 11,105 | 591,970 |  | 8,707 | 170,719 |  | 11,073 | 590,544 |
| 15 | 8,866 | 458,028 |  | 7,288 | 126,124 |  | 8,831 | 457,042 |
| 16 | 365 | 24,373 |  | 208 | 6,815 |  | 364 | 24,115 |
| 17 | 8,078 | 399,364 |  | 5,938 | 110,273 |  | 8,052 | 397,858 |
| 18 | 8,203 | 443,445 |  | 6,695 | 136,205 |  | 8,170 | 441,881 |
| 19 | 7,592 | 379,328 |  | 6,130 | 114,447 |  | 7,560 | 377,931 |
| 20 | 8,176 | 536,862 |  | 6,400 | 149,361 |  | 8,142 | 535,375 |
| 21 | 7,545 | 263,037 |  | 5,790 | 74,203 |  | 7,536 | 262,235 |
| 22 | 3,830 | 134,487 |  | 2,877 | 29,045 |  | 3,819 | 133,808 |
| 23 | 5,694 | 229,334 |  | 4,364 | 66,870 |  | 5,684 | 228,639 |
| 24 | 6,591 | 253,920 |  | 4,877 | 68,965 |  | 6,570 | 253,187 |
| 25 | 1,891 | 88,224 |  | 1,400 | 24,985 |  | 1,883 | 87,779 |
| 26 | 5,221 | 218,594 |  | 4,054 | 64,660 |  | 5,201 | 217,868 |
| 27 | 4,643 | 230,351 |  | 3,375 | 70,815 |  | 4,633 | 228,932 |
| 28 | 4,511 | 190,787 |  | 3,268 | 51,345 |  | 4,509 | 190,038 |
| Z | 22,494 | 2,381,301 |  | 11,764 | 356,841 |  | 22,421 | 2,369,317 |

^1^ Combined: the combined reference panel; ^2^ Internal, the internal reference panel; ^3^ External: the external reference panel; ^4^ Tar.snp: number of SNPs of tar panel; ^5^ Ref.snp: number of SNPs of reference panel.

**Supplementary Table S3.** **Number of markers of imputed WGS data and 600 K chip data with different R-squared cutoffs of linkage disequilibrium**

| R2 | Number of SNPs | |
| --- | --- | --- |
|  | 600 K^1^ | WGS^2^ |
| 1 | 547,020 | 7,582,487 |
| 0.99 | 371,334 | 1,535,966 |
| 0.9 | 270,552 | 1,286,797 |
| 0.8 | 223,474 | 1,102,272 |
| 0.7 | 180,364 | 925,829 |
| 0.6 | 140,512 | 762,992 |
| 0.5 | 104,362 | 612,191 |
| 0.4 | 73,130 | 477,306 |
| 0.3 | 47,762 | 357,503 |
| 0.2 | 28,932 | 253,756 |
| 0.1 | 16,403 | 171,324 |

^1^ 600 K array: the 600 K Affymetrix® Axiom® HD genotyping array.

^2^WGS: whole genome sequencing data

**Supplementary Table S4. Average number of selected variants from different p-valve cutoffs of GWAS results**

| Traits^1^ | Average number of selected variants | | | |
| --- | --- | --- | --- | --- |
|  | p-value>=2 | p-value>=3 | p-value>=4 | p-value>=5 |
| ADG | 888,006 | 336,158 | 94,340 | 21,758 |
| ADFI | 1,080,264 | 533,686 | 205,436 | 64,456 |
| RFI | 1,023,973 | 460,534 | 151,125 | 40,896 |
| FCR | 724,023 | 213,856 | 50,643 | 11,251 |
| CW | 799,977 | 256,008 | 55,910 | 10,312 |
| EWG | 807,916 | 267,266 | 60,260 | 11,548 |
| EW | 779,544 | 246,734 | 53,944 | 10,481 |
| BMW | 749,385 | 241,326 | 58,091 | 11,620 |
| DW | 688,080 | 174,845 | 31,843 | 4,523 |
| AFW | 842,417 | 297,128 | 75,934 | 1,4249 |
| AFP | 777,703 | 253,105 | 58,783 | 1,0076 |
| GW | 657,739 | 175,563 | 34,251 | 5,681 |
| IL | 764,083 | 239,878 | 54,704 | 11,326 |
| BW45 | 690,509 | 189,297 | 39,855 | 8,249 |
| BW49 | 696,879 | 183,927 | 32,759 | 5,409 |
| BW56 | 665,197 | 173,961 | 31,299 | 4,564 |
| BW63 | 667,908 | 177,052 | 32,748 | 5,472 |
| BW70 | 736,721 | 218,167 | 44,538 | 7,617 |
| BW77 | 809,159 | 259,130 | 57,657 | 11,019 |
| BW84 | 813,383 | 270,044 | 61,216 | 12,048 |
| BW91 | 780,753 | 239,238 | 51,972 | 10,006 |

^1^ These traits were average daily gain (ADG), average daily feed intake (ADFI), residual feed intake (RFI), feed conversion ratio (FCR), carcass weight (CW), breast muscle weight (BMW), eviscerated weight with giblets (EWG), eviscerated weight (EW), drumstick weight (DW), abdominal fat weight (AFW), abdominal fat percentage (AFP), gizzard weight (GW), intestine length (IL), body weight in 45 days (BW45), body weight in 49 days (BW49), body weight in 56 days (BW56), body weight in 63 days (BW63), body weight in 70 days (BW70), body weight in 77 days (BW77), body weight in 84 days (BW84), and body weight in 91 days (BW91).

**Supplementary Table S5. Average number of selected and remaining variants after different LD-based marker pruning**

| Variants | R2 | ADG | IL | BMW | RFI | BW91 | ADFI |
| --- | --- | --- | --- | --- | --- | --- | --- |
| selected | 0.1 | 33,849 | 24,582 | 24,508 | 48,035 | 24,332 | 55,087 |
|  | 0.2 | 51,370 | 37,452 | 37,313 | 72,561 | 36,982 | 83,410 |
|  | 0.3 | 74,185 | 53,913 | 53,523 | 103,958 | 53,206 | 119,800 |
|  | 0.4 | 101,159 | 73,314 | 72,576 | 141,037 | 72,322 | 162,490 |
|  | 0.5 | 131,703 | 95,317 | 94,061 | 182,901 | 93,932 | 210,929 |
|  | 0.6 | 165,896 | 120,028 | 118,307 | 229,743 | 118,206 | 265,149 |
|  | 0.7 | 203,070 | 146,492 | 144,764 | 280,659 | 144,554 | 324,042 |
|  | 0.8 | 243,206 | 174,918 | 173,703 | 334,636 | 173,090 | 387,406 |
|  | 0.9 | 284,299 | 204,084 | 203,884 | 390,585 | 202,131 | 452,155 |
|  | 0.99 | 336,158 | 239,878 | 241,326 | 460,534 | 239,238 | 533,686 |
| remaining | 0.1 | 137,946 | 147,099 | 147,183 | 48,035 | 147,356 | 116,798 |
|  | 0.2 | 203,378 | 217,110 | 217,240 | 72,561 | 217,536 | 171,616 |
|  | 0.3 | 284,692 | 304,656 | 304,989 | 103,958 | 305,406 | 239,512 |
|  | 0.4 | 378,043 | 405,497 | 406,185 | 141,037 | 406,535 | 317,304 |
|  | 0.5 | 482,623 | 518,586 | 519,790 | 182,901 | 520,002 | 404,147 |
|  | 0.6 | 599,503 | 644,856 | 646,599 | 229,743 | 646,723 | 501,072 |
|  | 0.7 | 725,304 | 781,340 | 783,093 | 280,659 | 783,341 | 605,230 |
|  | 0.8 | 861,729 | 929,444 | 930,699 | 334,636 | 931,349 | 718,500 |
|  | 0.9 | 1005,257 | 1,084,888 | 1,085,125 | 390,585 | 1,086,909 | 838,441 |
|  | 0.99 | 1202,533 | 1,298,209 | 1,296,752 | 460,534 | 1,298,897 | 1,006,028 |

*ADG: average daily gain; IL: intestine length; BMW: breast muscle weight, RFI: residual feed intake; BW91: body weight in 91 days; ADFI: average daily feed intake (ADFI)

**Supplementary Table S6. Regression coefficient of GBLUP of 21 traits in chicken using different variants selected from GWAS results**

| Traits^1^ | Regression coefficient (±S.E) | | | |
| --- | --- | --- | --- | --- |
|  | p-value>=2 | p-value>=3 | p-value>=4 | p-value>=5 |
| ADG | 17.65±0.01 | 1.09±0.01 | 0.72±0.01 | 0.51±0.01 |
| ADFI | 10.44±0.01 | 1.31±0.01 | 0.96±0.01 | 0.75±0.01 |
| RFI | 3.82±0.01 | 1.13±0.01 | 0.88±0.01 | 0.67±0.01 |
| FCR | 19.86±0.01 | 0.98±0.01 | 0.57±0.01 | 0.41±0.01 |
| CW | 0.92±0.01 | 0.74±0.01 | 0.54±0.01 | 0.45±0.01 |
| EWG | 0.88±0.01 | 0.72±0.01 | 0.52±0.01 | 0.40±0.01 |
| EW | 0.85±0.01 | 0.69±0.01 | 0.50±0.01 | 0.42±0.01 |
| BMW | 0.96±0.01 | 1.45±0.01 | 0.72±0.01 | 0.46±0.01 |
| DW | 1.24±0.01 | 1.21±0.01 | 0.50±0.01 | 0.33±0.01 |
| AFW | 8.30±0.01 | 1.16±0.01 | 0.68±0.01 | 0.48±0.01 |
| AFP | 10.78±0.01 | 1.01±0.01 | 0.59±0.01 | 0.45±0.01 |
| GW | 7.10±0.01 | 1.21±0.01 | 0.66±0.01 | 0.41±0.01 |
| IL | 17.76±0.01 | 1.09±0.01 | 0.73±0.01 | 0.57±0.01 |
| BW45 | 5.34±0.01 | 1.12±0.01 | 0.66±0.01 | 0.50±0.01 |
| BW49 | 2.82±0.01 | 1.00±0.01 | 0.57±0.01 | 0.45±0.01 |
| BW56 | 2.5±0.01 | 1.28±0.01 | 0.58±0.01 | 0.47±0.01 |
| BW63 | 1.39±0.01 | 1.54±0.01 | 0.68±0.01 | 0.54±0.01 |
| BW70 | 1.83±0.01 | 1.24±0.01 | 0.62±0.01 | 0.50±0.01 |
| BW77 | 1.94±0.01 | 1.13±0.01 | 0.67±0.01 | 0.53±0.01 |
| BW84 | 4.93±0.01 | 1.16±0.01 | 0.61±0.01 | 0.45±0.01 |
| BW91 | 277.49±0.01 | 1.14±0.01 | 0.61±0.01 | 0.51±0.01 |

^1^ These traits were average daily gain (ADG), average daily feed intake (ADFI), residual feed intake (RFI), feed conversion ratio (FCR), carcass weight (CW), breast muscle weight (BMW), eviscerated weight with giblets (EWG), eviscerated weight (EW), drumstick weight (DW), abdominal fat weight (AFW), abdominal fat percentage (AFP), gizzard weight (GW), intestine length (IL), body weight in 45 days (BW45), body weight in 49 days (BW49), body weight in 56 days (BW56), body weight in 63 days (BW63), body weight in 70 days (BW70), body weight in 77 days (BW77), body weight in 84 days (BW84), and body weight in 91 days (BW91).
